# Influence of Human Eating Habits on Antimicrobial Resistance Phenomenon: Aspects of Clinical Resistome of Gut Microbiota in Omnivores, Ovolactovegetarians, and Strict Vegetarians

**DOI:** 10.3390/antibiotics10030276

**Published:** 2021-03-09

**Authors:** Suzane Fernandes da Silva, Isabela Brito Reis, Melina Gabriela Monteiro, Vanessa Cordeiro Dias, Alessandra Barbosa Ferreira Machado, Vânia Lúcia da Silva, Cláudio Galuppo Diniz

**Affiliations:** Laboratory of Bacterial Physiology and Molecular Genetics, Center for Studies in Microbiology, Department of Parasitology, Microbiology and Immunology, Federal University of Juiz de Fora, 36036-330 Juiz de Fora, Brazil; sfs.fernandes@ufjf.br (S.F.d.S.); isabritoreis@ufjf.br (I.B.R.); melinamoreira84@gmail.com (M.G.M.); vanessa.dias@icb.ufjf.br (V.C.D.); alessandra.machado@icb.ufjf.br (A.B.F.M.); vania.silva@icb.ufjf.br (V.L.d.S.)

**Keywords:** clinical resistome, gut microbiota, eating habits, omnivorism, ovolactovegetarianism, strict vegetarianism

## Abstract

The use of xenobiotics in food production and how food intake is carried out in different cultures, along with different eating habits (omnivorism (ON), ovolactovegetarianism (VT), and strict vegetarianism (VG)) seem to have implications for antimicrobial resistance, especially in the human gut microbiota. Thus, the aim of this study was to evaluate aspects of the clinical resistome of the human gut microbiota among healthy individuals with different eating habits. Volunteers were divided into 3 groups: *n* = 19 omnivores (ON), *n* = 20 ovolactovegetarians (VT), and *n* = 19 strict vegetarians (VG), and nutritional and anthropometric parameters were measured. Metagenomic DNA from fecal samples was used as a template for PCR screening of 37 antimicrobial resistance genes (ARG) representative of commonly used agents in human medicine. The correlation between eating habits and ARG was evaluated. There were no significant differences in mean caloric intake. Mean protein intake was significantly higher in ON, and fiber and carbohydrate consumption was higher in VG. From the screened ARG, 22 were detected. No clear relationship between diets and the occurrence of ARG was observed. Resistance genes against tetracyclines, β-lactams, and the MLS group (macrolides, lincosamides, and streptogramins) were the most frequent, followed by resistance genes against sulfonamides and aminoglycosides. Vegetables and minimally processed foods seem to be the main source of ARG for the human gut microbiota. Although eating habits vary among individuals, the open environment and the widespread ARG from different human activities draw attention to the complexity of the antimicrobial resistance phenomenon which should be addressed by a One Health approach.

## 1. Introduction

The clinical resistome is defined as the set of genes related to antimicrobial resistance to drugs commonly used to treat infectious diseases in a particular environment [1,2]. The human gut microbiota is assumed to be an important reservoir of antimicrobial resistance genes (ARG), since it is always in contact with environmental microorganisms, which are introduced by oral contamination through food and water ingestion, and by contact with animals and other people. Horizontal transference of ARG may occur in the intestinal environment among commensal microorganisms, including potentially pathogenic ones, and pathogens [3,4].

In light of the One Health concept, in which human health is linked to animal and environmental health [5], the microbiomes of humans, animals, and the different environments show a connection with each other, allowing ARG to be shared and widely distributed [6,7]. According to the historic perspective, the One Health concept has focused on local interconnections and interdependencies. As a contemporary point of view, this approach considers global health and includes the comprehension of characteristics that may stimulate the global antimicrobial resistance (AMR) [6].

Human activities may induce changes in the open environment and exert an important selective pressure on microbial communities, favoring the spread of ARG and contributing to the maintenance of, and even increase in, the antimicrobial resistance phenomenon [8,9].

It is accepted that the food production chain may play an important role in the selection and spread of ARG over the open environment, with food commensal or contaminant microorganisms being recognized as sources of ARG [4,10]. Although some studies show the role of food microorganisms in the spread of ARG with impacts on the human gut resistome, the influence of different eating habits on the phenomenon is still poorly understood [11,12,13,14,15]. Therefore, the objective of this study was to comparatively evaluate aspects of the clinical resistome of the gut microbiota considering different eating habits, namely, omnivorism (ON), ovolactovegetarianism (VT), and strict vegetarianism (VG), and to investigate how food intake may contribute to resistome quality.

## 2. Results

The sociodemographic, anthropometric, and nutritional data of the participants are presented in Table 1. The average age of the participants was within the inclusion criteria and was overall 28.66 (±7.79) years old. Regarding ethnic groups, 72.4% were White and 19.0% were Pardo. Less than 10% belonged to other ethnic groups (Black and East Asian—6.9% and 1.7%, respectively), and the average BMI was 21.90. With regards to the gender distribution, 25.9% were male and 74.1% were female. No significant differences were observed in daily caloric intake; however, the lipid consumption was higher in ON compared with VG. Regarding protein intake, consumption was different between the three groups, being higher in omnivores. Carbohydrate consumption was significantly higher among VG than in the other two groups, and there were no significant differences between ON and VT. Regarding the consumption of total fibers, insoluble fibers, and soluble fibers, the three groups behaved differently, with fiber consumption in VG being higher than in the other two groups, and the eating behavior of ON participants showing the lowest fiber consumption.

**Figure 1:**
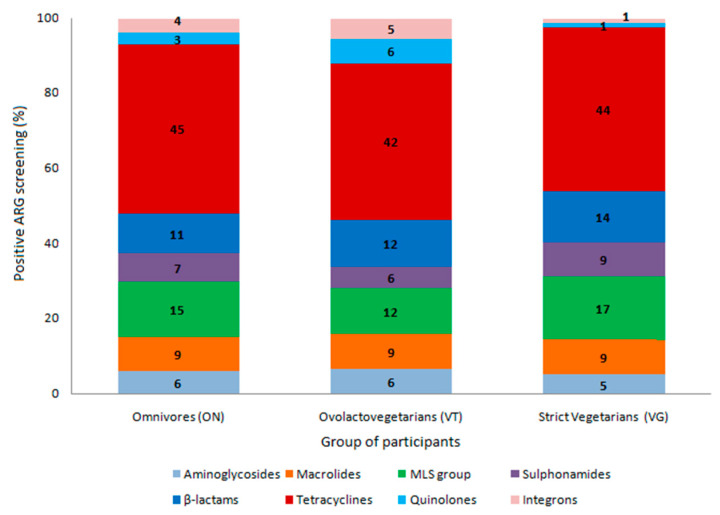
Frequency of detection of antimicrobial resistance genetic markers (ARG) related to different classes of antimicrobial drugs according to positive screening by PCR from the fecal metagenome of omnivores (ON), ovolactovegetarians (VT), and strict vegetarians (VG). ARG are clustered based on their chemical structure or phenotype, such as β-lactams (*bla_CTX-M_*, *bla_TEM_*, *bla_SHV_*, *blaZ*, *mef*); tetracyclines (*tet(A)*, *tet(B)*, *tet(E)*, *tet(L)*, *tet(M)*, *tet(O)*, *tet(Q)*); macrolide, lincosamide, and streptogramin (MLS) group (*ermA*, *ermB*, *ermC*); quinolones (*qnrB*, *qnrS*); sulfonamides (*sul1*, *sul2*); aminoglycosides (*aacA-aphD*); and integrons (*intl-1*, *intl-2*).

**Figure 2:**
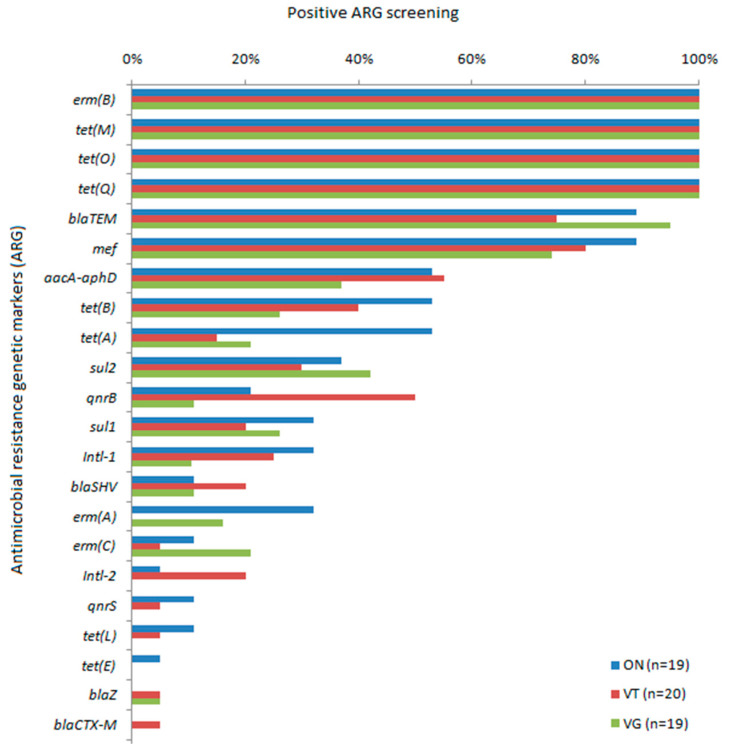
Distribution of 22 antimicrobial resistance genetic markers (ARG) detected out of 37 tested by PCR from the fecal metagenome of omnivores (ON), ovolactovegetarians (VT), and strict vegetarians (VG). Frequency of positive screening among each tested sample.

**Figure 3:**
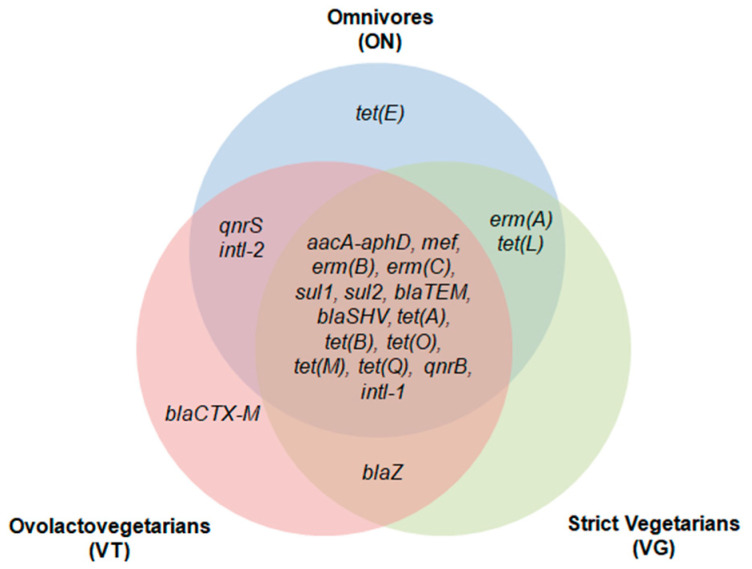
Venn diagram representing the occurrence of antimicrobial resistance genetic markers (ARG) and qualitative clustering according to their positive screening exclusively or shared between omnivores (ON), ovolactovegetarians (VT), and strict vegetarians (VG). β-lactams (*bla_CTX-M_*, *bla_TEM_*, *bla_SHV_*, *blaZ*, *mef*); tetracyclines (*tet(A)*, *tet(B)*, *tet(E)*, *tet(L)*, *tet(M)*, *tet(O)*, *tet(Q)*); macrolide lincosamide and streptogramin (MLS) group (*ermA*, *ermB*, *ermC*); quinolones (*qnrB*, *qnrS*); sulfonamides (*sul1*, *sul2*); aminoglycosides (*aacA-aphD*); and integrons (*intl-1*, *intl-2*).

From the 37 tested ARG, representative of the clinical resistome considering the most widely used antimicrobials in human medicine, 22 were detected at least in one group of individuals. Among the ARG detected, tetracycline resistance genes were the most observed among the three groups, followed by macrolide, lincosamide, and streptogramin (MLS) and macrolide resistance genes and β-lactam resistance genes (Figure 1). The *tet(M)*, *tet(Q)*, and *tet(O)* genes and *erm(B)* were detected in all samples analyzed. *bla_TEM_* and *mef* were detected at a frequency higher than 70% in the three groups. *aacA-aphD* was also frequently detected, ranging from 37% in VG to 55% in VT. The sulfonamide resistance genes, *sul1* and *sul2*, quinolone resistance genes, *qnrB* and *qnrS*, and *intl-1* genes had intermediate detection frequencies (Figure 2). Fifteen common markers were observed among all evaluated groups. ARG such as *qnrS* and *intl-2* were found only in ON and VT, while *erm(A)* and *tet(L)* were specifically detected in ON and VG; *blaZ* was observed in VT and VG, whereas *bla_CTX-M_* was detected in a single sample from the VT group and *tet(E)* detected in only one sample from the ON group (Figure 3).

**Figure 4:**
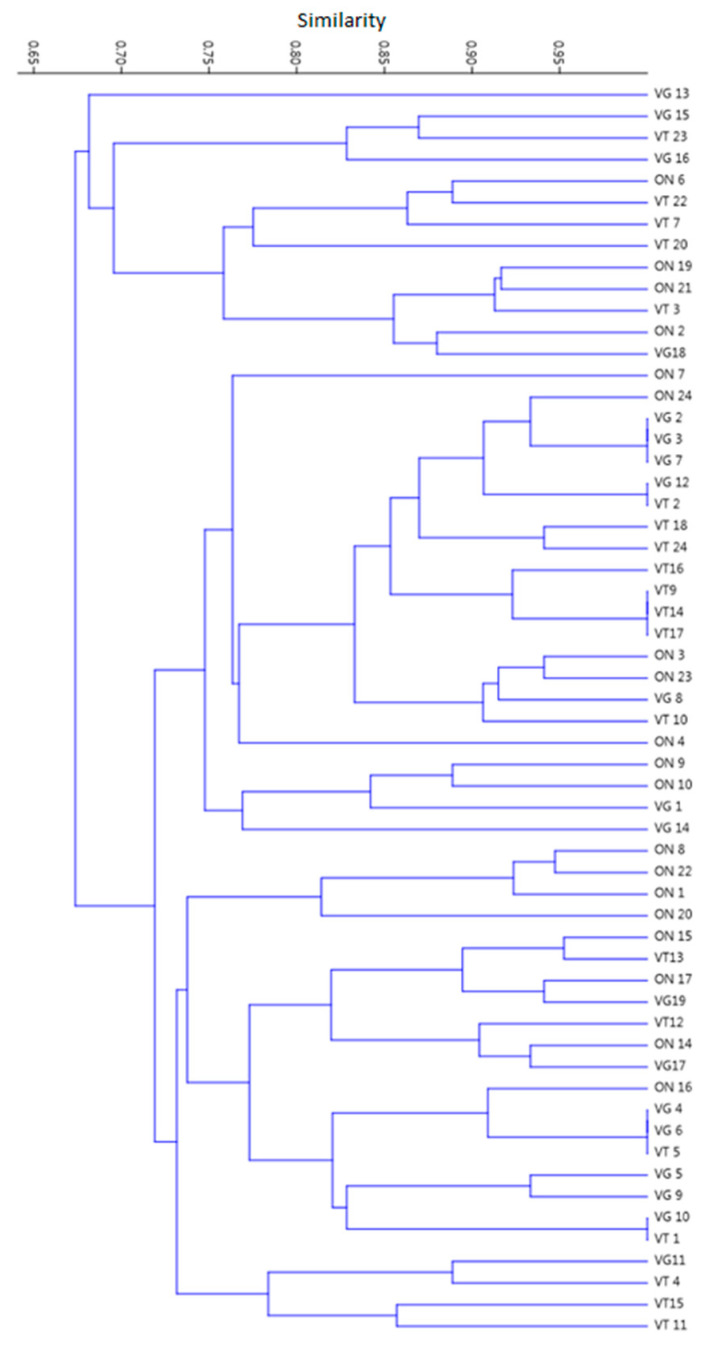
Clustering analysis of 58 healthy individuals with different eating habits (omnivores (ON), ovolactovegetarians (VT), and strict vegetarians (VG)) related to polymerase chain reaction (PCR) screening of antimicrobial resistance genes (ARG), representative of the human gut clinical resistome. The dendrogram was obtained from a binary matrix built based on positive or negative amplification of each tested ARG, using the Unweighted Pair Group Method with Arithmetic Mean (UPGMA) grouping method.

Taking together the ARG screening data considering the three different groups of individuals with specific eating habits, a highly homogeneous distribution of detected genetic markers was observed among the ON, VT, and VG. No significant clustering was observed in the similarity matrix and dendrogram obtained. Although two clusters were observed considering a threshold of similarity between 65% and 70%, no differentiation was observed within each cluster considering the participants in the study. The data support the observation that different eating habits may not influence the distribution of ARG in the human gut microbiota from a holistic point of view (Figure 4).

## 3. Discussion

Recently, issues related to antimicrobial resistance have changed as scientific information is gained regarding the origin, transmission, and evolution of the phenomenon. However, the interplay among humans, animals, and the environment in this context, including human activities, has not been completely elucidated [6]. Geography and behavior are among the key factors related to the human microbiota structure, although the microbial core is specific in different species [7,8]. In this regard, biotic and abiotic environmental factors are accepted to modulate a microorganism’s distribution in different ecosystems, especially considering the One Health approach. It is expected that the One Health approach will modify concepts to improve our understanding of ARG transmission and guide strategies to mitigate AMR [6]. Anthropogenic activities and habits such as eating behavior, culture, practices of antimicrobial use in different activity levels, sanitation, hygiene, and waste management have a impact on microbiota diversity as well as on ARG distribution, especially in the gut microbiota [8,16,17]. In addition, it is accepted that environmental resistomes may share a common core which is available to humans and other animals [6,18].

In this longitudinal and prospective study with a probabilistic sample of an average population representative of healthy Brazilians, all of them adult residents in urban areas with different eating habits, no significant differences were observed in the anthropometrical data, clearly supporting that nutritional habits were more likely related to eventual alterations in intestinal microbiota structure, more specifically the gut microbiota resistome. Considering eventual bias related to ethnic groups and their social behavior, it is difficult to address any discussion as most of the participants belonged to the White ethnic group followed by Pardo. The term Pardo is a particularly complex one and is used in Brazil to refer to people of mixed ethnic ancestries and social behavior [19]. Considering the other populational characteristics such as healthy status and being a resident of the same urban area, no differences were observed considering ethnicity. The observed predominance of fiber intake in VT and VG diets compared with ON may suggest that foods with a lower degree of processing, especially vegetables, may be considered the main source of ARG in these individuals, with a low impact of food from animal origin on microbial resistome quality. However, according to the literature, and as observed in the wide distribution of ARG, it is reasonable to suggest an interchange among human, animal, and plant microbiomes, as ARG related to antimicrobials used in veterinary medicine were detected in the gut resistome of VG individuals [6,7]. 

As for ON, foodstuffs of animal and plant origin are likely to contribute to the resistome composition. In contrast, for VT and VG, it is highly suggested that vegetable-associated microorganisms are the main source for ARG to build the gut resistome. It is important to reinforce that our data showed an overall similar final composition in ON, VT, and VG gut clinical resistomes. A homogeneous distribution of volunteers’ samples was observed with no clustering related to eating habits. From a different point of view, some authors state that overall resistome composition is not significantly impacted by diet; however, they suggest that meat and animal-derived foods may carry more ARG [7,20]. It must be stressed that in these reports geographical locations are different, and the numbers of screened ARG are lower than in our study along with inclusion and exclusion criteria, which may explain the non-uniform information drawn from the data.

On the other hand, ARG can remain stable in the human gut microbiota for long periods, even in the absence of antimicrobial selection [21]. Thus, ARG diversity is due not only to the direct effect of the selective pressure exerted by antimicrobial use, but also to transference of ARG between different microbiomes. Several studies have already highlighted the role of fecal contamination in the process of ARG spread, showing high prevalence of ARG in animal and human feces, sewage waste, and water treatment plants. Through soil and irrigation water, ARG may reach vegetables [6,22,23].

The plant microbiome shares ARG with the surrounding environment, and soil is probably the main ARG source for the resistome in plants [24]. As vegetables are usually eaten raw or with low processing, the chances of antimicrobial resistant bacteria and ARG remaining viable and reaching the human gut microbiota are greater [24,25,26]. Several studies have shown that organic vegetables have a richer resistome when compared with conventional ones, suggesting that the consumption of organic vegetables is also an important source of ARG for the human microbiota [27,28,29,30]. 

Tetracyclines, macrolides, β-lactams, and sulfonamides are among the most used antimicrobials in farm animals [31,32]. They can be used to treat infections, for prophylaxis, metaphylaxis, and, in low doses, as growth promoters. In Brazil, the use of several antimicrobials as growth promoters was banned in the early 2000s. However, the consumption of these drugs remains high, and it is estimated that in Brazil and in the other BRICS countries, antimicrobial consumption will double in livestock activities by the year 2030. Despite the ban, use of prophylaxis and metaphylaxis remains high [33,34].

ARG such as *tet(Q)* and *tet(O)* are frequently detected in farm animal feces and are shown to be a stable part of their gut resistome. These *tet* genes are commonly associated with transposons and mobile genetic elements, which may contribute to their wide distribution among environments [35]. Variants such as *tet(Q)*, *tet(O)*, and *tet(M)* are highly frequent genes in the human gut resistome [22]. It is known that *tet(Q)* is highly prevalent among isolates of *Bacteroides* spp., major anaerobic bacilli resident in the human gut microbiota. As for *erm* genes, they are also commonly found in healthy individuals, showing homology to alleles in different species, which may suggest horizontal spread [35,36,37]. Still, according to the literature, *tet* transcripts could play several additional roles, such as cell signaling, communication, or transport. Besides, toxic heavy metals and antimicrobials of the MLS group could participate in co-selection of tetracycline resistance [22,38,39]. 

The MLS group of ARG is usually detected in the environment related to human and animal microbiota, soil, water, and sewage [18,23,40]. Among them, *erm(B)* is the most common and highly frequent in the human gut microbiota and is associated with mobile genetic elements [18,23,40,41,42]. As for *mef*, also highly detected in the present study, they encode efflux pumps, and are commonly associated with transposons [42,43].

*bla_TEM_*, an important extended-spectrum beta-lactamase (ESBL) encoding gene, was the main beta-lactamase-related ARG detected in this study, corroborating other authors who describe *bla_TEM_* in human feces, animal origin foodstuffs, and sewage [31,44,45,46]. The *bla_TEM_* genes, together with other ARG related to macrolide, aminoglycoside, and tetracycline resistance, are accepted to be widely spread [18]. In fact, the *aacA-aphD* gene, reported to be an important aminoglycoside ARG, was also among the most observed in this study. 

Although some reports have described lower observation of *bla_TEM_* and higher detection of *bla_CTX-M_* and variants in human and animal feces and foodstuffs [20,47,48,49], the low *bla_CTX-M_* detection in this study raises some considerations: ARG persistency in each environment should be directly impacted by antimicrobial use and ESBL-producing bacteria epidemiology, including geography, movement of people, trade, and social behavior [50]. In Brazil, high detection rates of *bla_TEM_* in the human gut resistome were recently reported in both eutrophic, overweight, and obese volunteers, which may reinforce the role of local epidemiology as a feature related to ARG persistency in different regions [31].

Sulfonamide and quinolone ARG were also observed but to a lesser extent when compared with tetracycline, MLS, and macrolide genes, with *sul1* and most of all *sul2* genes being detected in all groups, considering the different eating habits. There is evidence of *sul* gene spread throughout water, soil, human and animal feces, vegetables, low processed foods, and sewage [32,51,52,53,54]. These antimicrobials, along with tetracyclines and penicillins, are often used in veterinary medicine. In addition, they seem to be highly persistent in soil, sediments, and water, due to properties such as high soil penetration and low removal efficiency, like tetracyclines [32]. 

Quinolone ARG such as *qnrB* and *qnrS* were also observed. Although the most common mechanism of quinolone resistance is chromosomal mutations, plasmid-mediated resistance can occur and is mainly related to *qnr* genes [55,56,57]. These genes confer low levels of resistance when compared to chromosomal resistance but may be horizontally transferred. In different ecossystems such as aquatic environments, livestock or human resistomes, more than one quinolone ARG may be observed in a mobile genome [55,56,58,59]. 

As described elsewhere, ARG are often associated with mobile genetic elements, mainly class 1, but also class 2 and 3 integrons [60]. In this study, *intl-1* was the most detected in all tested groups, in higher frequencies for ON and VT. Although *intl-2* was also detected, but at a lower frequency, it was not observed in the fecal metagenome of VG participants. Facing the frequency of observation and the core of ARG among the three groups of participants, it is reasonable to suggest that although integrons play an important role in ARG spread by horizontal transference, especially class 1, other mobile genetic elements, such as transposons and plasmids, also have an important role in ARG transfer in the gut microbiota. Further prospective studies are thus needed to map and better address the ARG transference routes in the gut microbiota.

## 4. Materials and Methods

### 4.1. Study Design

This a descriptive cross-sectional study with healthy volunteers, *n* = 58, resident in the southeast region of Brazil, with different eating habits (ON, *n* = 19; VT, *n* = 20; and VG, *n* = 19), invited to participate. The inclusion criteria were: (i) age ranging between 18 and 60 years old; (ii) dietary behavior for at least one year; (iii) body mass index (BMI) between 18.5 and 24.9; (iv) no antimicrobial agent intake in the past 3 months; (v) no pregnancy or breastfeeding; and (vi) no chronic diseases such as systemic pathologies, diabetes, hypertension, cancer, and rheumatoid arthritis. The study was approved by the Human Research Ethics Committee of the Federal University of Juiz de Fora. All volunteers were informed about the study and signed the Free and Informed Consent Form.

### 4.2. Participants’ Anthropometric Data and Dietary Assessment

Volunteers’ weight was measured on a digital scale in a central position, erect, barefoot, with feet together, and wearing as little clothing as possible. Height was measured by means of a vertical stadiometer attached to the scale, with the volunteer also barefoot and erect, with heels together, with no headdress and looking at the horizon. BMI was calculated based on the relationship between weight and height (Kg/m²) and evaluated according to the method proposed by the World Health Organization [61]. To reduce variation, the measures were always measured by the same person, using the same tape measure and scale.

Regular food intake was accessed by a quantitative food frequency questionnaire (QFFQ), according to the literature [62]. In these questionaries, the volunteers recorded the average food intake (daily, weekly, and monthly) in the past six months. A literature-validated photographic album was used to estimate food portion sizes [63].

### 4.3. Fecal Specimen Collection and Storage

After dietary assessment, stool samples were collected by spontaneous demand, fresh in its native state in a screw-cap sterile container, and transported to the laboratory within two hours in an ice box to be processed for metagenomic DNA extraction from 200 mg aliquots. Reminiscent stool samples were stored in a freezer at –80 °C. 

### 4.4. Extraction, Quantitation, and Integrity of Metagenomic DNA

The metagenomic DNA was extracted using the commercial kit QIAamp^TM^Fast DNA Stool Mini Kit (Qiagen, Hilden, Germany), according to the manufacturer’s instructions. Further, extracted DNA was eluted in a volume of 50 µL and kept in a freezer at –20 °C for further analysis. Metagenomic DNA quantification and purity were determined by the A260/A280 absorbance ratio with Nanodrop (Thermo Scientific NanoDrop^TM^2000 micro volume spectrophotometer, Thermo Fisher Scientific, Waltham, MA, USA).

DNA integrity was assessed by electrophoresis on 0.8% agarose gel in 0.5X TBE buffer (Tris-HCl-Borate-EDTA). The gel was stained with ethidium bromide and analyzed in an ultraviolet light transilluminator (GE Healthcare, Amersham, UK).

### 4.5. Screening of Antimicrobial Resistance Genes (ARG)

The clinical resistome assessed in this study consists of a set of 37 ARG, including classes 1, 2, and 3 integrons (Table S1, Supplementary Data). These resistance markers were PCR screened from metagenomics with specific primers and amplification conditions as previously described in the literature (Table S1, Supplementary Data).

The PCR reactions were performed in 25 µL containing 12.5 µL of PCR Master Mix (Promega, Madison, WI, USA) and 1 µL of DNA (~20 ng/µL). Primer volumes varied according to the ARG, and the total volume was completed with water. The reactions took place in an automated thermocycler (Biometra T1 Thermocycler, Gttingen, Germany), according to different running protocols, specific for each primer set (Table S1, Supplementary Data). After PCR reaction, electrophoresis was performed on 1.0% agarose gel, analyzed after ethidium bromide staining on an ultraviolet transilluminator (GE Healthcare, Amersham, UK). A molecular weight standard 1Kb DNA ladder (Promega, Madison, WI, USA) was used. Positive and negative controls were used for each PCR reaction.

### 4.6. Statistical Analysis

Student’s *t*-test was used to compare the macronutrient intake from the QFFQ, with a significance level of 5% (*p* = 0.05) and using the XLSTAT statistical program. To evaluate the clinical resistome similarity clustering according to the presence or absence of ARG detection, a dendrogram was constructed based on Dice similarity coefficient and UPGMA method (Unweighted Pair Group Method with Arithmetic Averages).

## 5. Conclusions

Human activities have a strong impact on microbiomes in different environments, with special concern for antimicrobial drug use. Although low processed foods might be considered an important source of ARG for resident microbiota, such as intestinal bacteria, there are several variables that may contribute to human gut resistome content which are still not entirely clear. In general, the results observed may suggest that vegetables and minimally processed foods are highly related to the core gut clinical resistome. In this study, healthy volunteers were screened for ARG from the fecal metagenome, with mostly different eating habits. On the other hand, from a One Health perspective, although vegetable and low processed food intake is thought to be the most common behavior in the sampled population, sources of ARG still may vary between different individuals. The ARG distribution in the environment seems to be so worrisome and widespread that eating habits may not be the only source of genetic elements and selective pressures that may impact the human gut resistome. Facing the relevance of antimicrobial resistance, further studies are needed to better address this issue in order to provide information support to the implementation of public and environmental policies to help to control the phenomenon.

## Figures and Tables

**Table 1:** Sociodemographic, anthropometric, and nutritional characteristics of the participants.

Characteristics		Group of Participants According to Their Eating Habits			*p* < 0.05 *
		ON (*n* = 19)	VT (*n* = 20)	VG (*n* = 19)	
Gender (%; male/female)		15.8/84.2	20.0/80.0	42.1/57.9	na
Average age (years ± SD)		28.47 ± 6.02	31.63 ± 9.72	25.89 ± 6.24	na
Ethnic Group (%)	White	52.6	78.9	89.5	na
	Pardo **	31.6	10.6	10.5	na
	Black	10.5	10.5		na
	East Asian	5.3			na
Average BMI (mean ± SD)		21.46 ± 1.96	22.13 ± 1.95	22.12 ± 1.72	
Mean daily calorie intake (% ± SD)		2049.3 ± 836.1	2092.4 ± 738.9	2522.7 ± 955.2	
Mean daily lipid intake (% ± SD)		33.39 ± 6.07	33.64 ± 14.41	25.10 ± 12.11	c
Mean daily protein intake (% ± SD)		19.22 ± 4.80	12.10 ± 3.16	10.17 ± 1.91	a, b, c
Mean daily CARB intake (% ± SD)		47.39 ± 7.35	54.26 ± 13.95	64.73 ± 11.34	b, c
Mean daily TF intake (g ± SD)		23.01 ± 9.04	41.98 ± 25.12	63.78 ± 35.79	a, b, c
Mean daily SF intake (g ± SD)		2.53 ± 1.56	4.98 ± 2.53	13.58 ± 14.65	a, b, c
Mean daily IF intake (g ± SD)		5.14 ± 3.27	8.88 ± 5.41	21.42 ± 19.36	a, b, c

* Significant statistical analysis, *p*-value was determined by Student’s *t*-test: a: comparison between ON and VT; b: comparison between VT and VG; c: comparison between ON and VG. ** The ethnic group Pardo is used in Brazil to refer to people of mixed ethnic ancestries and represents a diverse range of ethnic backgrounds. BMI = Body Mass Index; CARB = carbohydrates; TF = total fibers; SF = soluble fibers; IF = insoluble fibers. ON = omnivores; VT = ovolactovegetarians; VG = strict vegetarians. SD = standard deviation. na = not applicable.

## Supplementary Materials

The following are available online at https://www.mdpi.com/2079-6382/10/3/276/s1, Table S1: Resistance genetic markers screened, initiator oligonucleotides, size of expected amplicons, and references.
