# Supplementary material for: Influence of Human Eating Habits on Antimicrobial Resistance Phenomenon: Aspects of Clinical Resistome of Gut Microbiota in Omnivores, Ovolactovegetarians, and Strict Vegetarians
Source: Antibiotics (Basel). 2021 Mar 9;10(3):276. doi: 10.3390/antibiotics10030276 (PMC8001852; doi:10.3390/antibiotics10030276)
Supplement: Supplementary file 1 [file antibiotics-10-00276-s001.pdf]

Table S1. Resistance genetic markers screened, initiator oligonucleotides, size of expected amplicons and references.

Marker	Resistance Mechanism	Targeted antimicrobials	Sequence (5′-3′)	Amplicon (bp)	Reference
bla$_{CTX-M}$	Enzymatic inactivation (β-lactamase)	β-lactams	ATG TGC AGY ACC AGT AAA G<br>GGT CAC CAG AAG GAG C	562	[1]
bla$_{KPC}$	Enzymatic inactivation (β-lactamase)	β-lactams	ATG TCA CTG TAT CGC CGT CT<br>TTT TCA GAG CCT TAC TGC CC	892	[1]
bla$_{SHV}$	Enzymatic inactivation (β-lactamase)	β-lactams	CTT TAC TCG CCT TTA TCG GC<br>TTA CCG ACC GGC ATC TTT CC	982	[1]
bla$_{TEM}$	Enzymatic inactivation (β-lactamase)	β-lactams	GTG CGC GGA ACC CCT ATT<br>TTA CCA ATG CTT AAT CAG TGA GGC	968	[1]
bla$_{OXA-23}$	Enzymatic inactivation (β-lactamase)	β-lactams	GAT CGG ATT GGA GAA CCA GA<br>ATT TCT GAC CGC ATT TCC AT	501	[2]
bla$_{OXA-51}$	Enzymatic inactivation (β-lactamase)	β-lactams	TAA TGC TTT GAT CGG CCT TG<br>TGG ATT GCA CTT CAT CTT GG	353	[2]
blaZ	Enzymatic inactivation (β-lactamase)	β-lactams	ACT TCA ACA CCT GCT GCT TTC<br>TGA CCA CTT TTA TCA GCA ACC	173	[3]
ampC	Enzymatic inactivation (β-lactamase)	β-lactams	CTG TTC GAG ATC GGC TC<br>CGG TAT AGG TCG CGA G	166	[4]
bla$_{SPM-1}$	Enzymatic inactivation (β-lactamase)	β-lactams	CCT ACA ATC TAA CGG CGA CC<br>TCG CCG TGT CCA GGT ATA AC	649	[5]
mecA	Modified target site	β-lactams	GTA GAA ATG ACT GAA CGT CCG ATA A<br>CCA ATT CCA CAT TGT TTC GGT CTA A	310	[6]

Table S1. Continuation.

Marker	Resistance Mechanism	Targeted antimicrobials	Sequence (5′-3′)	Amplicon (bp)	Reference
tet(A)	Efflux pump	Tetracyclines	GCT ACA TCC TGC TTG CCT TC<br>CAT AGA TCG CCG TGA AGA GG	210	[7]
tet(B)	Efflux pump	Tetracyclines	TTG GTT AGG GGC AAG TTT TG<br>GTA ATG GGC CAA TAA CAC CG	659	[7]
tet(E)	Efflux pump	Tetracyclines	AAA CCA CAT CCT CCA TAC GC<br>AAA TAG GCC ACA ACC GTC AG	278	[7]
tet(K)	Efflux pump	Tetracyclines	GTA GCG ACA ATA GGT AAT AGT<br>GTA GTG ACA ATA AAC CTC CTA	360	[8]
tet(L)	Efflux pump	Tetracyclines	TCG TTA GCG TGC TGT CAT TC<br>GTA TCC CAC CAA TGT AGC CG	267	[7]
tet(M)	Ribosomal protection	Tetracyclines	AGT GGA GCG ATT ACA GAA<br>CAT ATG TCC TGG CGT GTC TA	158	[8]
tet(O)	Ribosomal protection	Tetracyclines	AGC GTC AAA GGG GAA TCA CTA TCC<br>CGG CGG GGT TGG CAA ATA	1723	[9]
tet(Q)	Ribosomal protection	Tetracyclines	TTA TAC TTC CTC CGG CAT CG<br>ATC GGT TCG AGA ATG TCC AC	904	[7]
msrA	Efflux pump	Macrolides/ streptogramins B	TCC AAT CAT AGC ACA AAA TC<br>AAT TCC CTC TAT TTG GTG GT	163	[10]
mef	Efflux pump	Macrolides	AGT ATC ATT AAT CAC TAG TGC<br>TTC TTC TGG TAC AAA AGT GG	348	[10]

Table S1. Continuation.

Marker	Resistance Mechanism	Targeted antimicrobials	Sequence (5′-3′)	Amplicon (bp)	Reference
erm(A)	Modified target site	MLS*	AAG CGG TAA ACC CCT CTG A<br>TTC GCA AAT CCC TTC TCA AC	190	[8]
erm(B)	Modified target site	MLS*	CTA TCT GAT TGT TGA AGA AGG ATT<br>GTT TAC TCT TGG TTT AGG ATG AAA	142	[3]
erm(C)	Modified target site	MLS*	AAT CGT CAA TTC CTG CAT GT<br>TAA TCG TGG AAT ACG GGT TTG	299	[8]
ere(A)	Enzymatic inactivation (erythromycin esterase)	Macrolides	AAC ACC CTG AAC CCA AGG GAC G<br>CTT CAC ATC CGG ATT CGC TCG A	420	[11]
ere(B)	Enzymatic inactivation (erythromycin esterase)	Macrolides	AGA AAT GGA GGT TCA TAC TTA CCA<br>CAT ATA ATC ATC ACC AAT GGC A	546	[11]
qnrB	Target site protection	Quinolones	GAT CGT GAA AGC CAG AAA GG<br>ATG AGC AAC GAT GCC TGG TA	476	[12]
qnrS	Target site protection	Quinolones	GCA AGT TCA TTG AAC AGG GT<br>TCT AAA CCG TCG AGT TCG GCG	428	[12]
sul1	Modified target site	Sulphonamides	ATG GTG ACG GTG TTC GGC ATT CTG A<br>CTA GGC ATG ATC TAA CCC TCG GTC T	815	[13]
sul2	Modified target site	Sulphonamides	CCT GTT TCG TCC GAC ACA GA<br>GAA GCG CAG CCG CAA TTC AT	396	[14]

* Macrolides, lincosamides and streptogramins

Table S1. Continuation.

Marker	Resistance Mechanism	Targeted antimicrobials	Sequence (5′-3′)	Amplicon (bp)	Reference
aacA-aphD	Enzymatic inactivation (acetyltransferase)	Aminoglycosides	TAA TCC AAG AGC AAT AAG GGC<br>GCC ACA CTA TCA TAA CCA CTA	227	[8]
mexB	Efflux pump	**	GTG TTC GGC TCG CAG TAC TC<br>AAC CGT CGG GAT TGA CCT TG	244	[15]
mexD	Efflux pump	**	CGA GCG CTA TTC GCT GC<br>GGC AGT TGC ACG TCG A	165	[4]
mexF	Efflux pump	**	CGC CTG GTC ACC GAG GAA GAG T<br>TAG TCC ATG GCT TGC GGG AAG C	255	[16]
mexY	Efflux pump	**	CCG CTA CAA CGG CTA TCC CT<br>AGC GGG ATC GAC CAG CTT TC	250	[15]
Intl1	Integration of genetic cassettes that may contain resistance genes	Class 1 Integron	GGT CAA GGA TCT GGA TTT CG<br>ACA TGC GTG TAA ATC ATC GTC	436	[17]
Intl2	Integration of genetic cassettes that may contain resistance genes	Class 2 Integron	CAC GGA TAT GCG ACA AAA AGG<br>TGTA GCA AAC GAG TGA CGA AAT G	788	[17]
Intl3	Integration of genetic cassettes that may contain resistance genes	Class 3 Integron	AGT GGG TGG CGA ATG AGT G<br>TGT TCT TGT ATC GGC AGG TG	600	[17]

** Efflux pumps that provide resistance to different antimicrobials

References:

1. Jones, C. H.; Tuckman, M.; Keeney, D.; Ruzin, A.; Bradford, P. A.; Characterization and sequence analysis of extended-spectrum-β-lactamase-encoding genes from *Escherichia coli*, *Klebsiella pneumoniae*, and *Proteus mirabilis* isolates collected during tigecycline phase 3 clinical trials. *Antimicrob. Agents Chemother.* **2009**, 53, pp. 465–475.
2. Woodford, N.; Ellington, M. J.; Coelho, J. M.; Turton, J. F.; Ward, M. E.; Brown, S.; Amyes, S. G. B.; Livermore, D. M. Multiplex PCR for genes encoding prevalent OXA carbapenemases in *Acinetobacter spp*. *Int. J. Antimicrob. Agents.* **2006**, 27, pp. 351–353.
3. Martineau, F.; Picard, F. J.; Lansac, N.; Ménard, C.; Roy, P. H.; Ouellette, M.; Bergeron, M. G. Correlation between the resistance genotype determined by multiplex PCR assays and the antibiotic susceptibility patterns of *Staphylococcus aureus* and *Staphylococcus epidermidis*. Antimicrob. *Agents Chemother.* **2000**, 44, pp. 231–238.
4. Xavier, D. E.; Picão, R. C.; Girardello, R.; Fehlberg, L. C.; Gales, A. C. Efflux pumps expression and its association with porin down-regulation and β-lactamase production among *Pseudomonas aeruginosa* causing bloodstream infections in Brazil. *BMC Microbiol.* **2010**, 10, pp. 217.
5. Gales, A. C.; Menezes, L. C.; Silbert, S.; Sader, H. S. Dissemination in distinct Brazilian regions of an epidemic carbapenem-resistant *Pseudomonas aeruginosa* producing SPM metallo-β-lactamase. *J. Antimicrob. Chemother.* **2003**, 52, pp. 699–702.
6. Zhang, K.; Sparling, J.; Chow, B. L.; Elsayed, S.; Hussain, Z.; Church, D. L.; Gregson, D. B.; Louie, T.; Conly, J. M. New quadriplex PCR assay for detection of methicillin and mupirocin resistance and simultaneous discrimination of *Staphylococcus aureus* from coagulase-negative Staphylococci. *J. Clin. Microbiol.* **2004**, 42, pp. 4947–4955.
7. Ng, L. K.; Martin, I.; Alfa, M.; Mulvey, M. Multiplex PCR for the detection of tetracycline resistant genes. *Mol. Cell. Probes* **2001**, 15, pp. 209–215.
8. Strommenger, B.; Kettlitz, C.; Werner, G.; Witte, W. Multiplex PCR assay for simultaneous detection of nine clinically relevant antibiotic resistance genes in *Staphylococcus aureus*. *J. Clin. Microbiol.* **2003**, 41, pp. 4089–4094.
9. Trzcinski, K.; Cooper, B. S.; Hryniewicz, W.; Dowson, C. G. Expression of resistance to tetracyclines in strains of methicillin-resistant Staphylococcus aureus. *J. Antimicrob. Chemother.* **2000**, 45, pp. 763–70.
10. Chaieb, K, Zmantar, T., Chehab, O., Boucham, O., Hasen, A. B., Mahdouani, K., & Bakhrouf, A. et al. Antibiotic resistance genes detected by multiplex PCR assays in *Staphylococcus epidermidis* strains isolated from dialysis fluid and needles in a dialysis service. *Jpn J Infect Dis.* **2007**, 60, pp. 183-187.
11. Sutcliffe, J.; Grebe, T.; Tait-Kamradt, A.; Wondrack, L. Detection of erythromycin-resistant determinants by PCR. *Antimicrob. Agents Chemother.* **1996**, 40, pp. 2562–2566.
12. Kim, H. B.; Park, C. H.; Kim, C. J.; Kim, E.-C.; Jacoby, G. A.; Hooper, D. C. Prevalence of plasmid-mediated quinolone resistance determinants over a 9-year period. *Antimicrob. Agents Chemother.* **2009**, 53, pp. 639–645.
13. Grape, M.; Sundström, L.; Kronvall, G. Sulphonamide resistance gene sul3 found in *Escherichia coli* isolates from human sources. *J. Antimicrob. Chemother.* **2003**, 52, pp. 1022–1024.
14. Hindi, A. K.; Shubbar, E. E.; Addos, S. A. Molecular study on distribution of sul-1 and sul-2 genes among *Salmonella enterica* causing enteric fever. *Mag. Al-Kufa Univ. Biol.* **2013**, 5, pp. 1–9.
15. Yoneda, K.; Chikumi, H.; Murata, T.; Gotoh, N.; Yamamoto, H.; Fujiwara, H.; Nishino, T.; Shimizu, E. Measurement of *Pseudomonas aeruginosa* multidrug efflux pumps by quantitative real-time polymerase chain reaction. *FEMS Microbiol. Lett.* **2005**, 243, pp. 125–131.
16. El Amin, N.; Giske, C. G.; Jalal, S.; Keijser, B.; Kronvall, G.; Wretlind, B. Carbapenem resistance mechanisms in *Pseudomonas aeruginosa*: alterations of porin OprD and efflux proteins do not fully explain resistance patterns observed in clinical isolates. *APMIS* **2005**, 113, pp. 187–196.

17. Machado, E.; Canton, R.; Baquero, F.; Galán, J-C.; Rollán, A.; Peixe, L.; Coque, T. M. Integron content of extended-spectrum-β-lactamase-producing *Escherichia coli* strains over 12 years in a single hospital in Madrid, Spain. *Antimicrob. Agents Chemother.* **2005**, 49, pp. 1823–1829.
